# Chitosan nanoparticle encapsulation increased the prophylactic efficacy of *Lactobacillus plantarum* RM1 against AFM_1_-induced hepatorenal toxicity in rats

**DOI:** 10.1007/s11356-023-31016-3

**Published:** 2023-11-23

**Authors:** Eman I. Hassanen, Lamiaa I. Ahmed, Karima M. Fahim, Mohamed G. Shehata, Ahmed N. Badr

**Affiliations:** 1 Department of Pathology, Faculty of Veterinary Medicine, Cairo University, Giza, 12211 Egypt; 2 Department of Food Hygiene and Control, Faculty of Veterinary Medicine, Cairo University, Giza, 12211 Egypt; 3 Department of Food Technology, Arid Lands Cultivation Research Institute, City of Scientific Research and Technological Application, Alexandria, Egypt; 4 Department of Food Toxicology and Contaminants, National Research Centre, Dokki, 12622 Cairo Egypt

**Keywords:** Aflatoxins M_1_, Chitosan nanoparticles, Hepatorenal toxicity, *Lactobacillus planterum*, Oxidative stress

## Abstract

Aflatoxin M_1_ (AFM_1_) is a significant contaminant of food, particularly dairy products and can resist various industrial processes. Several probiotic strains like *Lactobacillus plantarum* are known to reduce aflatoxin availability in synthetic media and some food products. The current work investigated the possible chitosan coating prophylactic efficacy of *Lactobacillus plantarum* RM1 nanoemulsion (CS-RM1) against AFM_1_-induced hepatorenal toxicity in rats. Twenty-eight male Wistar rats were divided into four groups (*n* = 7) as follows: group 1 received normal saline, group 2 received CS-RM1 (1mL contains 6.7 × 10^10^ CFU), group 3 received AFM_1_ (60 µg/kg bwt), and group 4 received both CS-RM1(1 mL contains 6.7 × 10^10^ CFU) and AFM_1_ (60 µg/kg bwt). All receiving materials were given to rats daily via oral gavage for 28 days. AFM_1_ caused a significant elevation in serum levels of ALT, AST, ALP, uric acid, urea, and creatinine with marked alterations in protein and lipid profiles. Additionally, AFM_1_ caused marked pathological changes in the liver and kidneys, such as cellular necrosis, vascular congestion, and interstitial inflammation. AFM_1_ also increased the MDA levels and decreased several enzymatic and non-enzymatic antioxidants. Liver and kidney sections of the AFM_1_ group displayed strong caspase-3, TNF-α, and iNOS immunopositivity. Co-treatment of CS-RM1 with AFM_1_ significantly lowered the investigated toxicological parameter changes and markedly improved the microscopic appearance of liver and kidneys. In conclusion, AFM_1_ induces hepatorenal oxidative stress damage via ROS overgeneration, which induces mitochondrial caspase-3-dependent apoptosis and inflammation. Furthermore, CS-RM1 can reduce AFM_1_ toxicity in both the liver and kidneys. The study recommends adding CS-RM1 to milk and milk products for AFM_1_-elimination.

## Introduction

Aflatoxins (AFs) are poisonous compounds produced by *Aspergillus* species, particularly *A. flavus* and *A. parasiticus* (Loi et al. 2020). AFB_1_ is metabolized by the hepatic microsomal cytochrome P450 and converted into AFM_1_ which is finally secreted into mammalian milk (Marchese et al. 2018). It is worth noting that adults, children, and infants extensively consume milk and its products. Kumar et al. (2016) revealed that more than 4.5 billion individuals worldwide will likely be exposed to foods contaminated with various amounts of AFs. Moreover, AFM_1_ is not destroyed once in milk and can withstand various industrial processes such as milk sterilization, pasteurization, and acidification. AFM_1_ contamination is still a significant issue in milk and all derivative products, such as cheese, yogurt, cream, and powdered milk (Ahmed et al. 2015; GadAllah et al. 2020). AFM_1_ produced carcinogenicity, genotoxicity, immunotoxicity, and cell injury to several organs (Min et al. 2020). AFM_1_ is mainly metabolized in the liver causing extensive hepatic toxicity, but its underlying mechanism is still unclear (Gao et al. 2022). Furthermore, AFB_1_ and AFM_1_ were associated with underweight children and neurologic impairment, with potentially increasing mortality rates (Marchese et al. 2018). However, research into the impact of AFM_1_ on kidney function and the mechanisms that underpin them is uncommon in lab animals.

Lactic acid-producing bacteria (LAB) are widely recognized for possessing physiological impacts on the intestine, including enhancing its activities and preserving human health (Fahim et al. 2023). LAB is broadly used to eliminate AFM_1_ from yogurt as it can bind various AFs in contaminated media (Gonçalves et al. 2020; Esam et al. 2022). However, it is incredibly susceptible to several factors such as heat treatment, air, bile salt solutions, and stomach PH. Therefore, the low gastric PH destroys most of the LAB that passes through the stomach after oral intake (Chen et al. 2020). Because the LAB has been shown to decrease in the gastrointestinal tract, bacterial protection with a controlled release could help mitigate AFM_1_ toxicity. Encapsulation is considered an excellent and effective solution for maintaining probiotics during processing and storage under adverse conditions without affecting their detoxification effect (Rodrigues et al. 2020). Control-released encapsulation systems can deliver probiotics to a specific target and release them at the appropriate time and place (Yoha et al. 2021).

Encapsulation substances, usually considered safe, can be utilized in food products and their edible film application (Badr et al. 2021). Applying natural polysaccharides for encapsulation does not require chemical solvents and can be performed at ambient temperature, representing a promising approach for encapsulating probiotics and supporting their ability for AFs binding (Abdel-Salam et al. 2020). Chitosan nanoparticles (CS NPs) have a high nutritional value and good sensory characteristics, in addition to their ability to supply functional components, such as self-assembly, small molecule bindings, excellent gelation properties, and complex formation interactions with other polymers (Hassanen et al. 2019a). Consequently, CS NPs are commonly used as a safe encapsulating material for several bioactive ingredients including LAB (Hassanen et al. 2021a).

Depending on the previous data, AFM_1_ has been becoming a global issue affecting human health, especially infants, who mainly depend on milk and milk products in their diet. Therefore, the present study investigated the mechanisms involved in AFM_1_-induced hepatorenal toxicity in rats, besides exploring the ameliorative effect of CS-RM1 nanoemulsion as a safe solution for reducing AFM_1_ toxicity.

## Materials and methods

### Chemicals, solvents, and reagents

Aflatoxin M_1_ standard, inulin, phosphate buffer saline (PBS), sodium alginate (viscosity 2000CPs; molecular weight (MW) 20,000 g/mol.; M/G ratio at 1.65), high MW chitosan (up to 80% deacetylation), sodium hydroxide, and glacial acetic acid were bought from Sigma Aldrich, Germany.

### Microorganisms, media, and cultivation conditions

The strain *Lactobacillus plantarum* RM1 was isolated from fermented Rayeb milk, locally purchased from a market in Alexandria, Egypt. The strain was reactivated first on De-Man Regosa and Sharp broth media (MRS; Conda, Spain) in 250 mL Erlenmeyer flasks. After activation, the strain was transferred to Lab-fermenters (Glass autoclavable Lab. Scale bioreactor, Lelesil Innovative Systems, Wagle Industrial Estate, Thane West, Thane, Maharashtra, India) containing the MRS-broth (37 °C/24 h). The yield was centrifuged at 4800 × g /20 °C/30 min to obtain the cell-pellets wealth of bioactive postbiotics; then, the cells were washed twice with a sterile peptone solution (0.1%). The next step was adding 1 g of bacterial pellet into phosphate buffer saline (1 mL/7.3 pH) to prepare 6.7 × 10^10^ CFU/mL concentration. The bacterial suspension was sonicated (15 min) to avoid cell precipitation and reduce the bacteria size. For improved homogeneity, drops of Tween 80 were added to the solution (Yeung et al. 2016).

### Preparation of bacterial encapsulation solution

To encapsulate the bacterial cells, two solutions were prepared to form nano-composite materials for further treatment. Solution (A) was prepared using sodium alginate (3%, w/v) in double distilled water using Erlenmeyer beaker (2L), according to the methodology described before (Moradi Pour et al. 2022). The solution was stirred carefully using a magnetic stirrer (700 rpm/1 h), and then, 1.5 g inulin was added carefully with continuous stirring. The second solution (B) was prepared using chitosan (2%, w/v), dissolved in double distilled water, and acidified using acetic acid (1%, v/v) using the methodology described by (Divya and Jisha 2018); the pH was adjusted at 6–6.2 using sodium hydroxide (1 M), stirred for 1 h, and filtered using Whatman No. 4 filter paper. (1%, v/v). Prepared solutions of (A) and (B) were autoclaved (121 °C/15 min) before mixing or bacterial loading.

Regarding the bacterial suspension preparation, a volume of the enrichment media (MRS) was inoculated by bacterial cells and incubated 16 h at 37 °C. By the end of the incubation, bacterial cells were harvested using centrifugation (3000 × g/ 4 °C/10 min) to get the bacterial pellet. The harvested pellet was re-suspended in calcium chloride solution before loading it into the alginate solution (Moradi Pour et al. 2022). The bacterial suspension was added to the solution (A) using a Hamilton syringe to ensure encapsulation occurrence during stirring using a mechanical stirrer (1 h/ 4 °C). The bacterial solution was loaded dropwise at a concentration of 6.7 × 10^10^ CFU/mL. The second solution was adding drops wisely to the complex of alginate loaded by bacteria, and the stirring process was completed for over 2 h. The resulting solution of encapsulated bacteria was 1.5 min stirred using an Ultra-Torexx homogenizer (30,000 rpm/min), followed by ultrasonication (Ultrasonic probe, processor UP400S, Hielscher Ultra-sound technology, USA) for 2.5 min. The encapsulated solution was then kept at 5 °C for further application.

### Characterization of the encapsulated bacteria

A dynamic light scattering instrument (Nano ZS, Malvern, Worcestershire, UK) was used to estimate the particle size distribution curve and zeta potential. The polydispersity index (PDI), which corresponds to the Stokes–Einstein relation, was also used to estimate the particle size as Z-average. Additionally, the particle shape was determined by transmission electron microscope (HR-TEM, Tecnai G20, Super twin, double tilt, FEI, Netherland).

### Animals and experimental design

Twenty-eight male albino Wistar rats weighing 150 ± 20 g were acquired from the lab animal house, National Research Center, Cairo, Egypt. Rats were reared in plastic pens with 4 or 3 rats per cage in a well-ventilated environment, receiving 12 h of light every day. The rats were fed a basal diet according to AIN-93 guidelines and provided with water ad-lib during the experimental period. The balanced diet was prepared to contain 21.6% protein supplemented from casein, 15% corn oil, 58.4% maize starch, 4% salt mixture, and 1% vitamin mixture. To make sure that all rats were in good health, they spent 2 weeks adapting to the place before the experiment began. All animal treatment procedures were followed the Cairo University’s Institutional Animal Care and Use Committee (approval No: Vet CU 09092023798) and the National Research Center’s Guide for Care and Use of Laboratory Animals (Publication No. 85–23, amended 1985), Cairo, Egypt.

Rats were randomly separated into four groups (*n* = 7). Group 1 was a control group that administered sterile normal saline daily throughout the experimental period. Group 2 administered CS-RM1 (1mL contains 6.7 × 10^10^ CFU). Group 3 administered AFM_1_ at dosage level of 60 µg/kg bwt. Group 4 co-administered CS-*RM1* with AFM_1_ at the same previously mentioned doses. All the materials mentioned above were administered to rats every day through the oral routes for 28 days. From the knowledge of the previous literature, there is no data explaining the LD50 of AFM_1_ in rats. However, one recent study indicated the LD50 of AFM_1_ in mice, which ranges from 9 to 16 mg/kg bwt (Güç et al. 2022). So, we used this paper to determine the AFM_1_-LD50 in rats (4.5–8 mg/kg bwt) utilizing the dose conversion formula between several experimental animal species, https://dosecal.cftri.res.in/index.php. Additionally, the AFM_1_-dosage level (1/100 LD50) employed in this investigation is that humans are probably exposed to daily via consuming contaminated milk and/or milk products. All rats were observed daily over the experimental period, and we recorded any clinical signs of illness if present.

### Sampling

After 28 days, rats were fasting overnight and anesthetized using ketamine (90 mg/kg) and xylazine (10 mg/kg) to collect blood samples via retro-orbital puncture; then, we centrifuged them at 3400 × g/10 min to obtain clear serum samples that preserved at − 20 °C till used for further applications. After blood sampling, rats were euthanized by cervical dislocation to obtain hepatic and renal tissue specimens. Some samples were stored at − 80 °C, while others were fixed in a 10% neutral buffer formalin solution.

### Biochemical parameter evaluation

Serum levels of urea, uric acid, creatinine, aspartate transaminase (AST), alanine transaminase (ALT), alkaline phosphatase (ALP), total protein (TB), albumin (ALB), total cholesterol (CHL), triglycerides (TG), and high-density lipoprotein concentrations (HDL) were determined in accordance with the manufacturer’s instructions for the kits (Biodiagnostic Co., Giza, Egypt). The concentrations of both low-density lipoproteins (LDL) and very low-density lipoproteins (VLDL) were estimated using the methods outlined by Friedewald et al. (1972). Moreover, the difference between the total proteins and albumin quantities was used to find the concentration of globulin (GLB).

### Oxidants/antioxidant estimation

Weighted tissue samples from the liver and kidneys were homogenized using a cold buffer (50 mM potassium phosphate buffer with 1 mM EDTA, pH 7.4) to assess some oxidants and antioxidant markers such as malondialdehyde (MDA)**,** catalase (CAT), superoxide dismutase (SOD), reduced glutathione (GSH), glutathione reductase (GR), and glutathione-S-transferase (GST) in accordance with the manufacturer’s instructions for the acquired kits (Biodiagnostic Com. Egypt).

### Histopathology

All the collected tissue specimens were processed using a standard procedure that utilized different concentrations of alcohol (70, 80, 90, 100) and xylene, afterward instilled in paraffin wax, cut at 4.5 μm, and colored by hematoxylin and eosin stain (H&E) to prepare stained slides ready to be examined under light Olympus microscope (Bancroft and Gamble 2013).

Seven random microscopic areas in each of seven sections, representing seven rats in each group were surveyed to assess the extent of the histological abnormalities in both hepatic and renal tissues as indicated by the strategy reported by Hassan et al. (2023). The pathological parameters for evaluating the hepatic alterations were hepatocellular degeneration, necrosis, inflammation, and fibroplasia. Tubular epithelial degeneration, necrosis, and interstitial hemorrhage were additional criteria utilized to evaluate the extent of renal damage. On a scale of 1 to 5, each observable lesion was rated and given a score of none, slight, mild, moderate, or severe changes (1 = normal histology, 2 < 25%, 3 = 25:50%, 4 = 50:75%, and 5 > 75% tissue damage) (Hassanen et al. 2021b).

### Tissue localization of some apoptotic and inflammatory immune markers

Caspase‐3, tumor necrosis factor-α (TNF-α), and inducible nitric oxide synthase (iNOS) were localized within hepatic and renal tissue using the avidin–biotin peroxidase method. The slides were first treated with primary antibodies against caspase-3, TNF-α, and iNOS (Abcam, Ltd.) at 1/200 dilutions, washed, and then incubated with peroxidase block (Sakura BIO) and a tool for detecting the presence of the antigen‐antibody complex (Power‐Stain 1.0 Poly HRP DAP Kit; Sakura). The sections were treated with diaminobenzidine-chromogen substrate, then counterstained by hematoxylin, and examined under an Olympus light microscope.

The protein expression of the studied immune markers was quantitatively analyzed using the Image J software by measuring the mean percentage area of brown positive immunostaining reaction relating to the total target area.

### Statistical analysis

Data were analyzed using the SPSS version 16.0 software (SPSS Inc, Chicago, IL) and represented as mean ± SEM. The one-way analysis of variance was done following the Tukey post hoc test. The threshold for statistical significance was *P* ≤ 0.05. Nonparametric values were represented as a median and analyzed using the Kruskal–Wallis *H*-test following the Mann–Whitney *U-*test.

## Results

### Characterization of the encapsulated bacteria

**Fig. 1:**
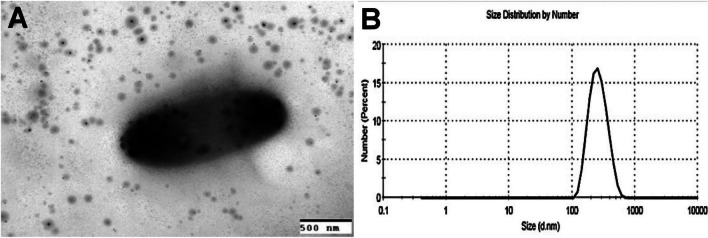
The size distribution curve (**A**) and HR-TEM image (**B**) of the prepared nanoemulsion

Chitosan nanoemulsion of RM1 bacterial solution was identified by a transmission electron microscope and showed spherical-shaped particles. The size of particles ranged between 536 and 741 nm; however, the inulin particle in the emulsion was recorded as more minor. The value of zeta potential was recorded at + 21.71 ± 0.84 mV, and the polydispersing index was 0.31 ± 0.03 (Fig. 1).

### Biochemical parameter evaluation

In contrast to the control group, a significant elevation in the serum levels of ALT, AST, ALP, urea, uric acid, and creatinine was recorded (Table 1). Meanwhile, the protein and lipid profile values of the AFM_1_ exposed group were significantly distorted in contrast to the control group. Otherwise, co-treatment of CS-RM1 with AFM_1_ reduced all of the above-mentioned biomarker levels. No significant differences were recorded in all parameters between the control group and those receiving CS-RM1 only.

**Table 1:** The effect of AFM_1_ and/or CS-RM1 on serum biomarker levels

	Control	CS-RM1	AFM_1_	AFM_1_ + CS-RM1
ALT (U/I)	33.7 ± 1.17	33.6 ± 1.24 ^a^	74.1 ± 1.97 ^b^	50.2 ± 1.54 ^a, b^
AST (U/I)	63.7 ± 1.81	63.4 ± 1.63 ^a^	99.8 ± 3.77 ^b^	81.1 ± 2.54 ^a, b^
ALP (U/I)	51.2 ± 1.44	51.4 ± 1.21 ^a^	94.8 ± 3.71 ^b^	72.4 ± 2.66 ^a, b^
TB (g/dL)	5.66 ± 0.21	5.74 ± 0.18 ^a^	4.28 ± 0.42 ^b^	4.37 ± 0.31 ^b^
ALB (g/dL)	3.51 ± 0.05	3.53 ± 0.02	2.81 ± 0.1	3.01 ± 0.09
GLB (g/dL)	1.46 ± 0.01	1.44 ± 0.02	1.05 ± 0.04	1.19 ± 0.1
CHL (mg/dL)	90.73 ± 3.47	91.24 ± 3.18 ^a^	181.54 ± 21.14 ^b^	132.22 ± 11.51 ^a, b^
TG (mg/dL)	64.7 ± 1.34	64.9 ± 1.21 ^a^	109.4 ± 6.45 ^b^	84.3 ± 2.74 ^a, b^
HDL (mg/dL)	32.7 ± 1.22	32.8 ± 1.14 ^a^	89.4 ± 3.17 ^b^	69.7 ± 2.34 ^a, b^
LDL (mg/dL)	54.6 ± 2.1	54.4 ± 2.23 ^a^	109.8 ± 4.91 ^b^	79.1 ± 2.18 ^a, b^
vLDL (mg/dL)	12.94 ± 0.27	12.98 ± 0.42 ^a^	21.88 ± 1.29 ^b^	16.86 ± 0.55 ^a, b^
Urea (mg/dL)	7.1 ± 0.02	7.3 ± 0.01 ^a^	9.84 ± 0.11 ^b^	8.14 ± 0.09 ^a, b^
UA (mg/dL)	1.7 ± 0.03	1.76 ± 0.04 ^a^	2.59 ± 0.17 ^b^	2.19 ± 0.14 ^a^
Creatinine (mg/dL)	0.7 ± 0.01	0.71 ± 0.02 ^a^	1.21 ± 0.11 ^b^	0.97 ± 0.13 ^a^

Values were expressed as mean ± SEM (*n* = 7). Within the same raw, the lowercase (a) means a significant difference from the AFM_1_ group, while (b) means a significant difference from the control group at *P* ≤ 0.05

### Oxidative stress evaluation

The AFM_1_ receiving group noticed a remarkable raise in MDA levels with a reduction in the content/activity of TAC, catalase, SOD, GSH, GST, and GR in both liver and kidneys compared with the control group. Otherwise, the group administered the CS-RM1 with AFM_1_ had a significant reduction of MDA levels and elevation of TAC, catalase, SOD, GSH, GST, and GR activity compared with the AFM_1_ receiving group; however, it is still distorted in comparison to that of the control group (Table 2).

**Table 2:** The effect of AFM_1_ and/or CS-RM1 on some oxidative stress markers in liver and kidneys

	Control	CS-RM1	AFM_1_	AFM_1_ + CS-RM1
Liver				
MDA (nmol/L)	43.4 ± 3.11	45.8 ± 1.88 ^a^	97.4 ± 2.87 ^b^	77.3 ± 4.18 ^a, b^
CAT (U/g)	75.6 ± 1.31	77.2 ± 1.41 ^a^	41.9 ± 1.63 ^b^	56.7 ± 1.64 ^a, b^
SOD (nmol/L)	1522.7 ± 21.8	1534.7 ± 43.3 ^a^	1699.8 ± 87.1 ^b^	1591.7 ± 72.4 ^a, b^
GST (U/g)	8.69 ± 1.31	8.73 ± 0.64 ^a^	8.11 ± 0.74 ^b^	8.49 ± 1.08 ^a^
GSH (mg/g)	13.6 ± 0.11	13.4 ± 0.21 ^a^	10.7 ± 1.37 ^b^	13.6 ± 0.11 ^a^
GR (U/g)	1.66 ± 0.14	1.71 ± 0.05 ^a^	0.81 ± 0.09 ^b^	1.08 ± 0.15 ^a, b^
Kidneys				
MDA (nmol/L)	8.4 ± 1.61	11.2 ± 2.81 ^a^	69.9 ± 4.66 ^b^	21.6 ± 2.66 ^a, b^
CAT (U/g)	24.8 ± 0.88	25.6 ± 1.05 ^a^	17.4 ± 1.54 ^b^	20.9 ± 1.19 ^a, b^
SOD (nmol/L)	1560.2 ± 161.7	1580.8 ± 67.4 ^a^	1421.1 ± 168.4 ^b^	1499.4 ± 89.9 ^a, b^
GST (U/g)	6.83 ± 0.43	6.91 ± 0.34 ^a^	4.68 ± 0.37 ^b^	4.47 ± 0.66 ^b^
GSH (mg/g)	49.4 ± 3.8	50.4 ± 2.74 ^a^	45.2 ± 1.16 ^b^	47.7 ± 1.31 ^a, b^
GR (U/g)	0.39 ± 0.05	0.38 ± 0.01 ^a^	0.24 ± 0.04 ^b^	0.29 ± 0.02 ^a^

Values were expressed as mean ± SEM (*n* = 7). Within the same raw, the lowercase (a) means a significant difference from the AFM_1_ group, while (b) means a significant difference from the control group at *P* ≤ 0.05

### Histopathological examination

**Fig. 2:**
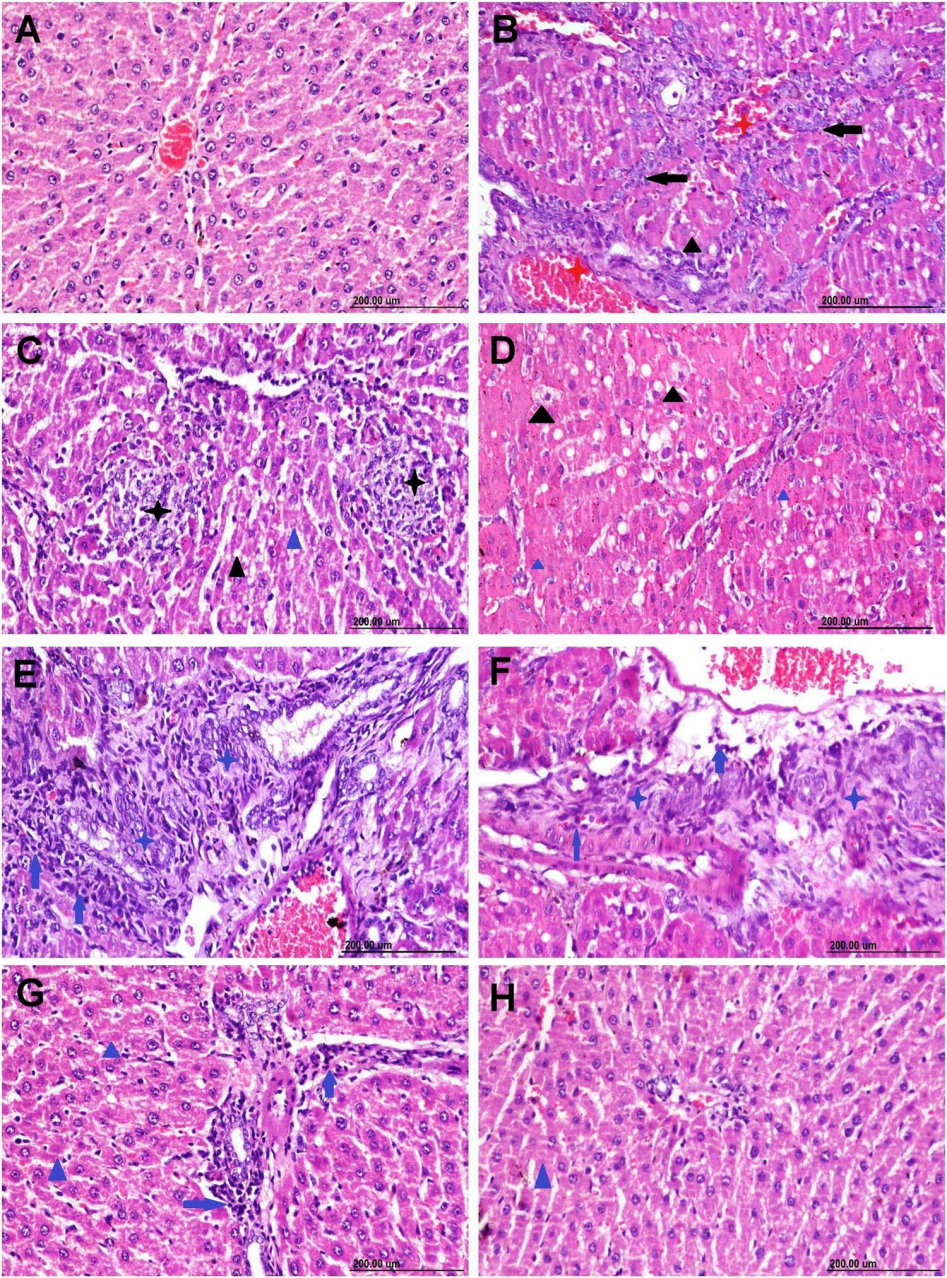
Hematoxylin and eosin stained hepatic photographs representing different experimental groups. **A** The control group exhibits normal histology. **B**–**F** The AFM_1_ group exhibits severe histological abnormalities. Note: oval cell hyperplasia (black arrow), focal coagulative necrosis (black star), hepatocellular vacuolization (black triangle) and necrosis (blue triangle), portal fibroplasia (blue star), portal inflammation (blue arrow). **G**–**H** The AFM_1_ + CS-RM1 group displayed sparse cell necrosis (blue triangle) with moderate portal inflammatory cell infiltration (blue arrow)

The microscopic picture of rats’ liver slices from the control group and CS-RM1 receiving group shows normal histological architectures (Fig. 2A). Sever histopathological alterations were observed in the group receiving AFM_1_. Most sections showed congestion in hepatic blood vessels and sinusoids along with extensive oval cell hyperplasia (Fig. 2B). There were random multifocal areas of hepatocellular necrosis extensively infiltrated with inflammatory cells (Fig. 2C). The majority of hepatocytes suffered from vacuolar degeneration and necrosis (Fig. 2D). The portal triad was extensively infiltrated with fibroblast and mononuclear inflammatory cells (Fig. 2E). Hyperplasia and dysplasia of epithelial lining bile ducts were recorded associated with newly formed bile ductulus and portal fibroplasia (Fig. 2F). Pretreatment of CS-RM1 with AFM_1_ markedly improved the histologic image of liver sections (Fig. 2G). Hepatocytes were intact with normal arrangements only individual cell necrosis and a slight increase in Kupffer cell numbers were recorded (Fig. 2H).

**Fig. 3:**
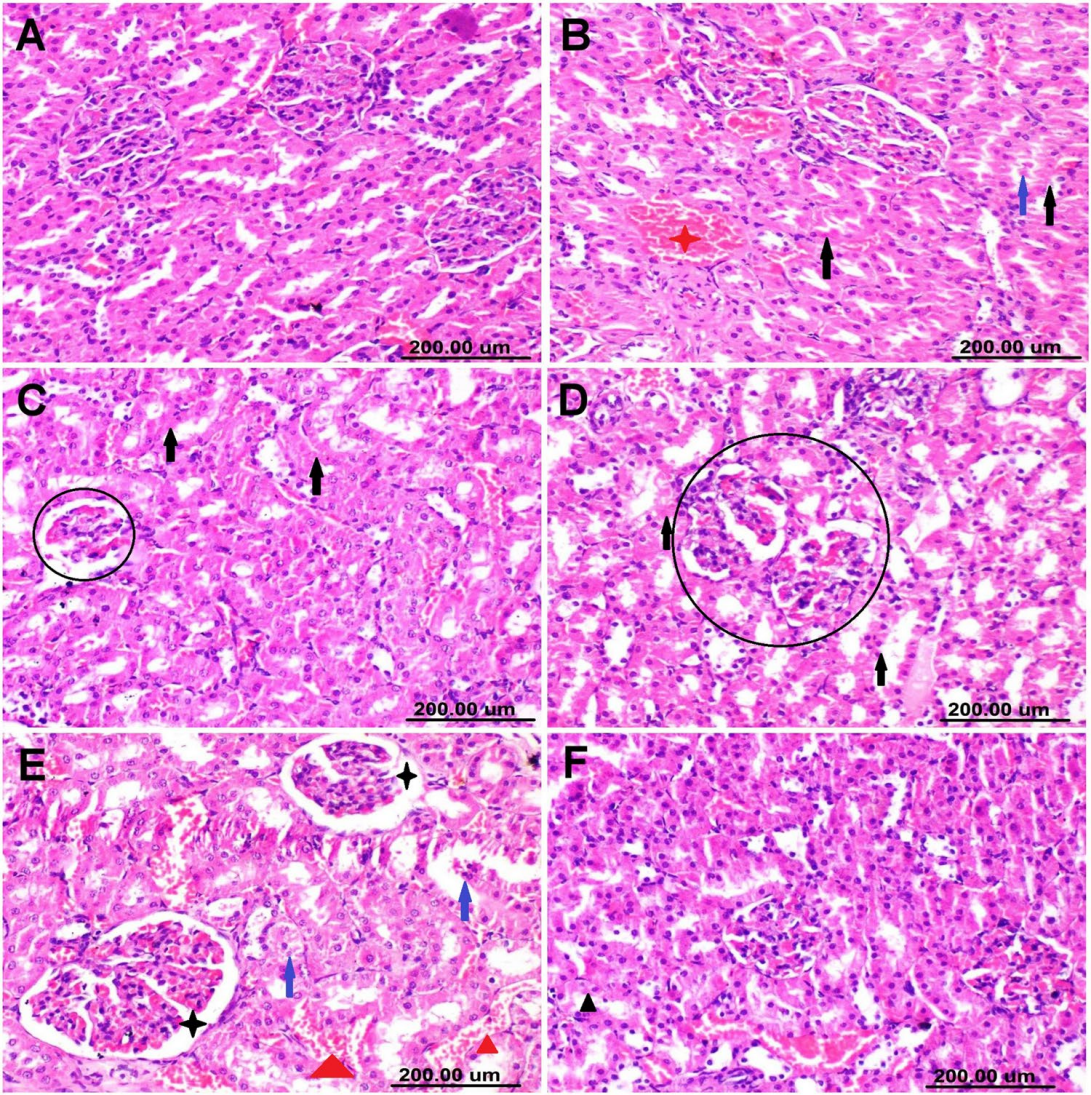
Hematoxylin and eosin-stained kidney photographs representing different experimental groups. **A** The control group exhibits normal histology. **B**–**C** The AFM_1_ group exhibits moderate histological abnormalities. Note: vascular congestion (red star), RBC extravasation (red triangle), cytoplasmic vacuolization (black triangle), necrosis (back arrow) and desquamation (blue arrows) of tubular epithelium, glomerular damage (circle), widening of bowman’s space (black star). **D** The AFM_1_ + CS-RM1 group displayed sparse vacuolar degeneration (black arrow) of tubular epithelium

Kidney sections of control rats and those receiving CS-RM1 showed typical histological structures **(**Figs. 3A). Kidneys of AFM_1_-receiving rats showed moderate pathological alterations. Most sections demonstrated interstitial congestion, cytoplasmic vacuolization, necrosis, and desquamation in some of the tubular epithelium (Fig. 3B). Besides the nephrotoxic nephrosis, some sections displayed mild to moderate glomerulopathy. Some glomeruli exhibit extensive damage, while others showed atrophy of the glomerular tuft with widening of Bowman’s space (Fig. 3C-E). Otherwise, pretreatment of AFM_1_-exposed rats with CS-RM1 significantly improved the microscopic picture of kidney sections which showed minimal tubular epithelial vacuolization and sparse cell necrosis (Figs. 3F).

**Fig. 4:**
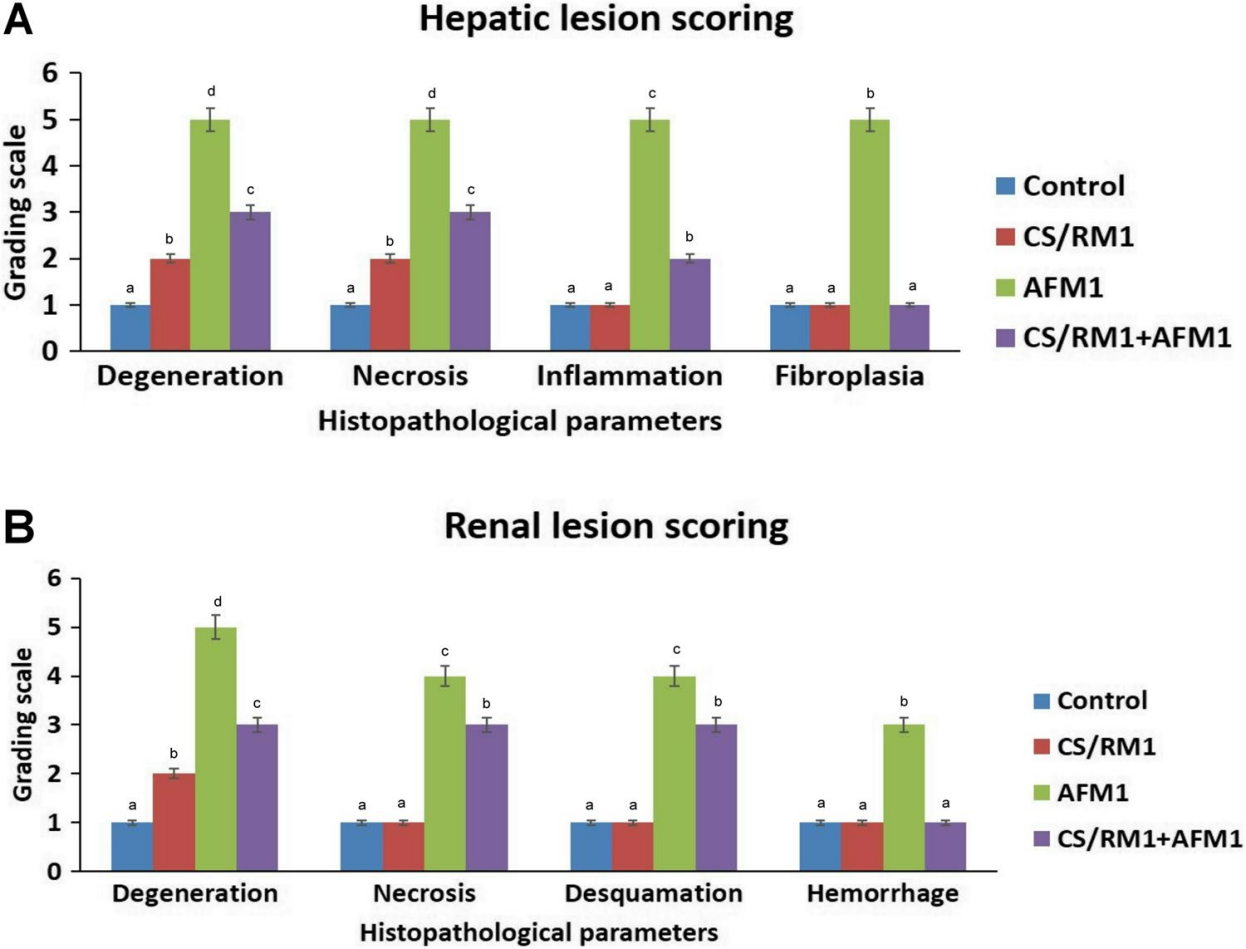
Bar chart representing the microscopic lesion scoring in the liver (**A**) and kidneys (**B**). Values were expressed as median (*n* = 35 random microscopic fields/group). Different uppercase letters (a, b, c, d) mean significant difference at *P* ≤ 0.05

The results of the microscopic lesion scoring are illustrated in Fig. 4. The highest score was recorded in the AFM_1_ group, while the AFM_1_ + CS-RM1 cotreated group demonstrated a significant decrease in all parameters’ scores in contrast to the AFM_1_ group.

### Immunohistochemical staining

**Fig. 5:**
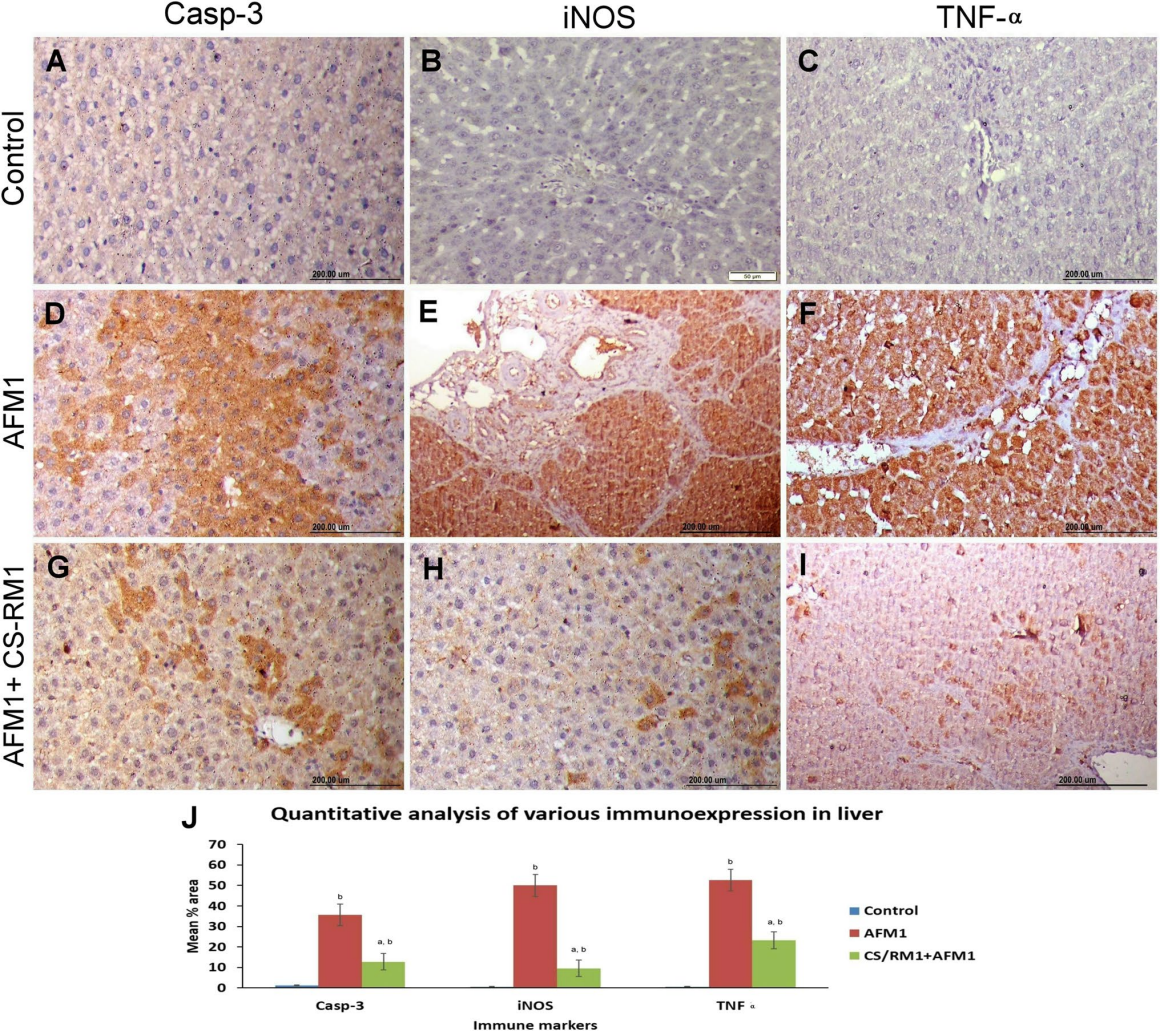
**A**–**I** Photographs representing the protein expression of caspase-3, iNOS, and TNF-α in the liver of different groups. **A**–**C** The control group showed negative expression of the studied immune markers. **D**–**F**) The AFM_1_ group displayed strong immunopositivity of the above-mentioned immune markers. **G**–**I** The AFM_1_ + CS-RM1 group displayed weak reaction for all immune markers. **J** Bar chart representing the mean percentage area of caspase-3, iNOS, and TNF-α immunostaining in the liver of different groups. Values were expressed as mean ± SEM (*n* = 35 random microscopic fields/group). The lowercase letter (a) means a significant difference from the AFM_1_ group, while (b) means a significant difference from the control group at *P* ≤ 0.05

**Fig. 6:**
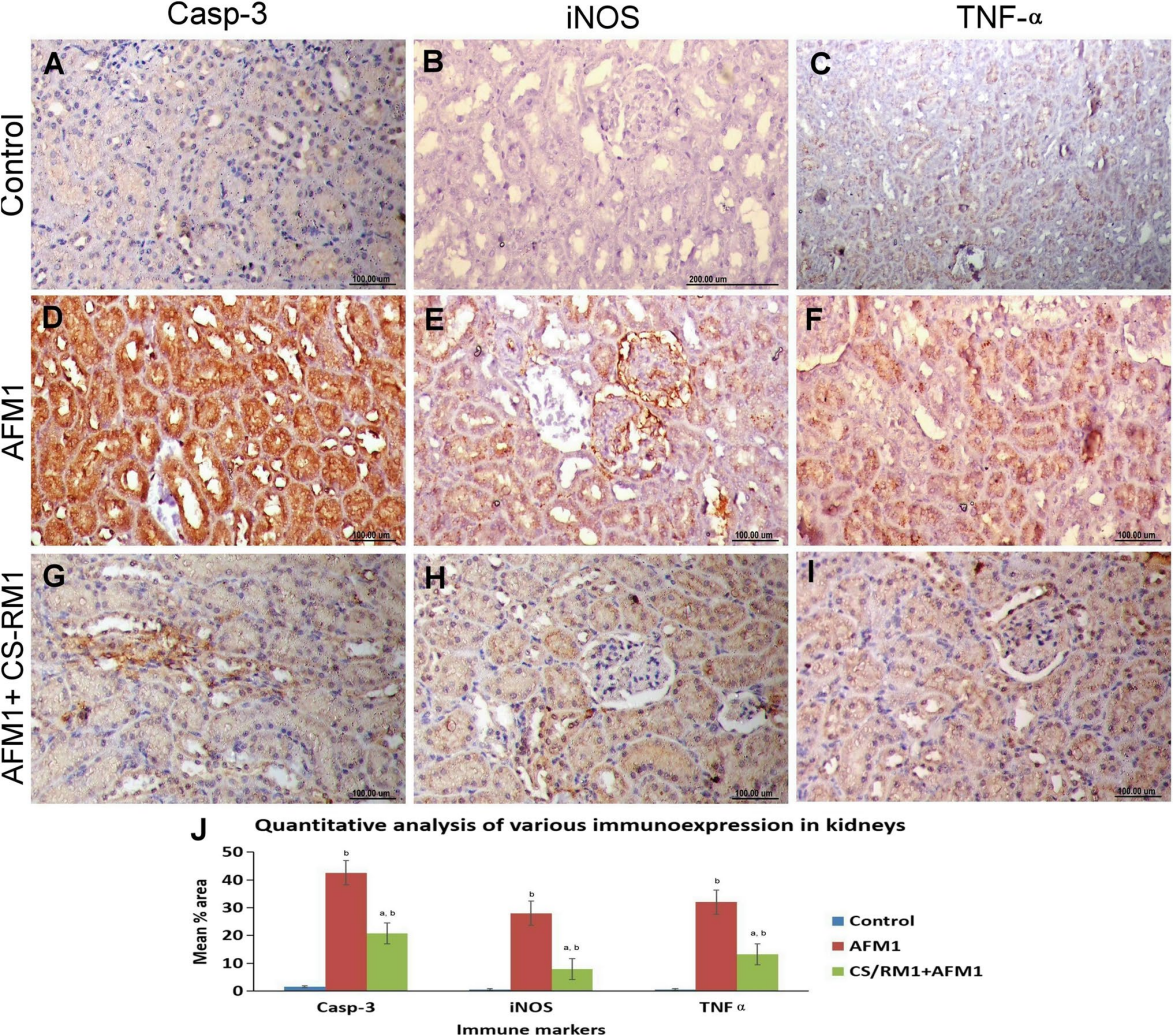
**A**–**I** Photographs representing the protein expression of caspase-3, iNOS, and TNF-α in kidneys of different groups. **A**–**C** The control group showed negative expression of the studied immune markers. **D**–**F** The AFM_1_ group displayed strong immunopositivity of the above-mentioned immune markers. **G**–**I** The AFM_1_ + CS-RM1 group displayed weak reaction for all immune markers. **J** Bar chart representing the mean percentage area of caspase-3, iNOS, and TNF-α immunostaining in the kidneys of different groups. Values were expressed as mean ± SEM (*n* = 35 random microscopic fields/group). The lowercase letter (a) means a significant difference from the AFM_1_ group, while (b) means a significant difference from the control group at *P* ≤ 0.05

The results of the immunohistochemical examination revealed potent caspase 3, TNF-α, and iNOS immunostaining reactions in both the liver and kidneys of rats receiving AFM_1_ compared with that of normal control. Co-treatment of CS-RM1 with AFM_1_ could reduce the immunomarker protein expression in both liver and kidney sections (Figs. 5 and 6).

## Discussion

AFM_1_ represents a global health issue for both industrialized and developing countries. Humans are repeatedly exposed to large quantities of AFM_1_ after consumption of contaminated foods including milk and dairy products (Stoloff et al. 2015; Wood 2018). Because of the strong thermal stability of AFM_1_, pasteurization of milk and other high thermal treatment (> 100 °C) could not degrade it, resulting in contamination of milk products with AFM_1_ (Iha et al. 2023). However, there were few in vitro studies regarding the toxicity of AFM_1_, and to better understand its effects on human health, more studies should be carried out on experimental animals.

In our investigation, serum levels of AST, ALT, ALP, creatinine, UA, and urea were significantly increased in the group exposed to AFM_1_, indicating hepatic and renal damage. Additionally, AFM_1_ caused alteration in both serum protein and lipid profiles of rats as a result of liver damage (site of protein synthesis and lipid metabolism). The histopathological examination confirmed these finding which showed severe cellular changes, including vacuolar degeneration and necrosis, along with widespread stromal inflammation. As a result of these histopathological changes induced by AFM_1_, enzymes leak from the damaged cells into the circulation, and their levels are increased. Together, these results confirmed that AFM_1_ could target the liver (site of detoxification and metabolism) and kidneys (site of excretion) in rats. It has been reported that AFM_1_ is a secondary metabolite of AFB_1_ formed in the liver by the action of CYP450 (Marchese et al. 2018). Accordingly, we suggested that the mechanism of AFM_1_-induced toxicity was similar to that of AFB_1_. Our findings were similar to those obtained by Li et al. (2019 & 2020), who confirmed the effect of AFB_1_ on liver and kidney biomarkers and microscopic pictures. Additionally, few researchers reported that AFM_1_ causes abnormal elevation in the serum levels of both liver and kidney biomarkers (Güç et al. 2022). All the biochemical and histopathological alterations noticed in the group receiving AFM_1_ may be attributed to AFM_1_-mediated oxidative stress.

In the current study, AFM_1_ could enhance the lipid peroxidation in both liver and kidney tissues, manifested by an increase in the levels of MDA and a decrease in both enzymatic (CAT, SOD, GST) and non-enzymatic (GSH, GR) antioxidants suggesting that AFM_1_-induced cytotoxicity could be attributed to the oxidative stress. MDA is a late indicator of oxidative cellular damage as it is a byproduct of lipid peroxidation (Khalaf et al. 2021). Oxidative stress is produced when the ROS generations exceed the capacity of the antioxidant scavenger (Morgan et al. 2021). GSH directly interacts with ROS and is considered an enzymatic detoxification cofactor; thus, it plays an essential role in cell protection against AFM_1_ exposure (Zhang et al. 2015; Azouz and Hassanen 2020). GSH depletion may be explained by its conjugation with AFM_1_ and/or its covalent binding with the reactive intermediates generated by the continuous attack of free radicals (Yilmaz and BAG 2022; Jabbar et al. 2023a). Furthermore, SOD being an antioxidant enzyme, it could protect various organisms against oxidative injury by converting the superoxide anion radicals to hydrogen peroxide. TAC indicates of the overall antioxidative activity reflecting the activity of all antioxidants in an organism (Asgary et al. 2022). Oxidative stress is implicated in several pathological disorders, including apoptosis, necrosis, and inflammation. ROS overgeneration leads to the exhaustion of both enzymatic and anti-enzymatic antioxidants causing lipid peroxidation, protein degradation, and organelles membrane damage. Additionally, AFM_1_ can induce cell membrane damage either directly through its toxic effect or indirectly via mitochondrial-dependent ROS generation (Zhang 2017; Ebedy et al. 2022a). This supports the hypothesis that oxidative stress is an important step in AFM_1_-induced hepatorenal damage**.**

Apoptosis is another mechanism implicated in AFM_1_-induced hepatorenal toxicity. It is well known that ROS production can activate the intrinsic mitochondrial-dependent pathway of apoptosis (Hassanen et al. 2019b). Our finding showed that the AFM_1_ group noticed widespread localization of caspase-3 protein throughout the hepatorenal tissues. AFM_1_-induced ROS generation, as previously discussed, increases the permeability of the mitochondrial membrane and opens the transition pores, resulting in cytochrome c release to the cytosol, which initiates activation of caspase cascade ends with caspase-3 activation (Mariod et al. 2023; Jabbar et al. 2023b). The caspase-3 is a protein member of the cysteine-aspartic acid protease family and is the end product of caspase activation (Hassanen et al. 2023a). It has an important role in the execution phase of programmed cell death. Additionally, the obtained findings proved that AFM_1_ could induce oxido-inflammatory stress via stimulation of some pro-inflammatory markers (iNOS) and cytokines (TNF-α). Moreover, nitric oxide (NO), produced due to iNOS activation, mediates several pathological processes, such as oxidative/nitrosative stress, cell death, and inflammation (Ebedy et al. 2022b; Hassanen et al. 2023b). Our immunohistochemical findings confirmed that AFM_1_ contributed to liver and kidney damage via the TNFα-signaling pathway that damaged the endothelial lining hepatic and renal blood vessels, resulting in edema, hemorrhage, and inflammation.

It is crucial to find organic adsorbents for AFM_1_ degradation without affecting the nutritional value of food. Recent studies focus on evaluating the binding capacity of different LAB strains with aflatoxins **(**Kuharić et al. 2018; Liu et al. 2020). Furthermore, nanotechnology is broadly applied in the food sector, such as adding nanoparticles, nanoemulsion, nanofibers, and nanoencapsulation of valuable biomaterials. It has been shown that compared to micro- or macro-sized components, nanoparticles provide a greater surface area for AFs bindings. By using LAB for AF-detoxification, it is directly added to the food and should be utilized in high amounts to perform their effect (Ceylan et al. 2016). Moreover, it cannot properly create a surface coating for food components with a larger surface area, making their usage in some food products ineffective (Ceylan et al. 2019). For these reasons, applying nanotechnology is a way to carry out unique strategies with diverse attributes. In our study, we prepared a nanoemulsion of CS-RM1 that was identified by HR-TEM and Zetasizer. The values recorded for the prepared nanoemulsion point out the better emulsion stability and the smaller size of inulin content. These characteristics can play an additional function in the in vivo applications during the rat’s experimental performance. The pretreatment of rats with a combination of CS-RM1 and AFM_1_ could significantly alleviate AFM_1_-mediated hepatorenal oxidative stress. CS-RM1 significantly improved the oxidant/antioxidant balance and returned the histological pictures of both liver and kidneys to nearly normal histology. So, it significantly normalized all the investigated serum biomarkers including lipid and protein profiles as well as liver and kidney biomarkers. CS-RM1 also reduced casp-3, TNF-α, and iNOS immunoexpression in the liver and kidneys.

Many researchers revealed the ability of LAB to degrade, eliminate, and bind to AFs; however, it reduced the bioavailability of AFs in humans and animals (Bangar et al. 2021). Furthermore, our previous work confirmed the strong binding capacity of RM1 with AFM_1_ and its in vitro antifungal activity against a wide range of fungi (Fahim et al. 2021). Additionally, several LAB strains, such as *P. pentosaceus* and *L. plantarum*, have a strong antioxidant capacity and protect many organs from ROS-inducing oxidative stress damage (Feng and Wang 2020). The prepared nanoformulation of CS-RM1 significantly ameliorated the AFM_1_-induced oxidative stress by reducing the MDA levels and elevating the content/activity of antioxidants, including GSH, GST, SOD, and CAT. Numerous in vitro investigations claimed that LAB strains have antioxidant characteristics and neutralize ROS through enzymatic pathways, such as catalase, superoxide dismutase, and a combined NADH oxidase/peroxidase system (Kong et al. 2021; Kim et al. 2022). By scavenging free radicals such as the hydroxyl radical or hydrogen peroxide, many pathological processes were prevented, such as lipid peroxidation, protein degradation, cellular apoptosis/necrosis, and tissue inflammatory reactions (Hassanen et al. 2023c). Superoxide dismutase and/or glutathione are excessively produced by the majority of LAB strains, which aid in removing extra ROS and protect both cell and mitochondrial membrane phospholipids from further damage (Bryukhanov et al. 2022). Based on this finding, LAB reduced the lipid peroxidation reaction, maintained the mitochondrial function, reduced the cytosolic [Ca2 +] levels, and regulated the inflammatory response via inactivating several inflammatory markers, such as iNOS and pro-inflammatory cytokines as TNF-α (Kim et al. 2021). Our results suggested the anti-inflammatory effect of CS-RM1 nanoemulsion manifested by weak or negative immunoexpression of both TNF-α and iNOS in both liver and kidneys. It is reported that LAB reduced TNF-α production by forming soluble molecules in activated macrophages (LeBlanc et al. 2020; Hassanen and Ragab 2020). Besides the overall advantage of LAB, we suggest that the nanoencapsulation of RM1 with CSNPs could enhance its solubility, dispersion, bioavailability, and stability, as well as control the release of RM1 to increase its efficiency (Bhattacharyya et al. 2016). Additionally, CS NPs displayed potent antioxidants (Mo et al. 2022), antiapoptotic, anti-inflammatory (Hassanen et al. 2022), and antimicrobial potentials (Hassanen and Ragab 2020). Our findings were in agreement with Trung and Bao (2015) who reported the antioxidant activity of chitosan via capturing the free radicals and protecting the cell membrane from lipid peroxidation. Additionally, it can regulate immunoinflammatory responses and promote the production of various anti-inflammatory cytokines, including IL-6, IL4, IL13, and IL10 (Fong and Hoemann 2017). Several studies revealed that CS NPs exerts anti-apoptotic effect via reducing ROS generations and inactivates several caspases as casp-3 and casp-9 (AbdElrazek et al. 2023).

The current study has many limitations, including a lack of molecular mechanisms, which must be explored for AFM_1_ toxicity and probiotic protection. In the present study, we used one dose only of AFM_1_ based on the oral LD50 of AFM_1_ in mice and there is a significant difference between rats and mice. Further studies required to detect the LD50 of AFM_1_ in rats as well as to investigate the toxicity of lower dosage levels of AFM_1_ than those reported in our study for a longer duration. Furthermore, we used a natural polymer CS NPs for encapsulating RM1. More studies required to investigate the potential of encapsulating probiotic with other coating materials, such as lipids, liposomes, and silica and spot the difference between them. Additionally, it is an important issue to determine the adsorbent efficacy of probiotic nanoemulsion by measuring AFs residues in all organs and frequently measuring its elimination in feces and urine. Our study opens a broad scope of research about the application of nanotechnology in food sectors as the encapsulating agent to control the release of probiotics to a specific target at the appropriate time and place.

## Conclusion

In conclusion, our results demonstrated that AFM_1_ induced hepatorenal toxicity through ROS overgeneration. It initiated hepatorenal oxidative stress damage and other correlating pathological processes such as apoptosis, necrosis, and inflammation. We also demonstrated that the CS-RM1 has a wide variety of modulatory qualities against AFM_1_-induced hepatorenal toxicity. CS-RM1 exerts potent antioxidant effects by decreasing the MDA levels and increasing both enzymatic and nonenzymatic antioxidant activity. CS-RM1 decreased the immune expression of caspase-3, iNOS, and TNF-α contributing to its anti-apoptotic and anti-inflammatory properties.

## Data Availability

All data are available on request.

## Declarations

All Institutional and National Guidelines for the care and use of animals were followed.
